# Evaluating consistency of physical activity and exercise prescription in the UK for people with diabetes – a Delphi study

**DOI:** 10.3389/fcdhc.2023.1278597

**Published:** 2023-12-07

**Authors:** Clare Strongman, Francesca Cavallerio, Matthew A. Timmis, Andrew Morrison

**Affiliations:** Cambridge Centre for Sport and Exercise Sciences, Anglia Ruskin University, Cambridge, United Kingdom

**Keywords:** diabetes, exercise, physical activity, public health, Delphi study

## Abstract

**Introduction:**

Increased physical activity is recommended as a cost-effective measure to tackle long-term management of people with diabetes, but research on interventions lacks consistency in terms of effective duration and modality.

**Methods:**

The aim of this study was to evaluate expert consensus on exercise and physical activity prescription via a three-round Delphi study conducted with 45 UK-based health and fitness professionals experienced in prescribing exercise or physical activity to people with diabetes.

**Results:**

The majority of items put forward to the panel reached consensus with 70% or above voting these items as important, but the details of the type, duration and/or modality of exercise or physical activity prescription within these items often contradicted each other, suggesting that patients are receiving inconsistent advice. The range of different exercise prescription found in this study suggests that patients are being given inconsistent and potentially confusing advice, which may affect their participation in exercise and long-term lifestyle change.

**Conclusion:**

More consistent promotion of advice from healthcare and fitness professionals may help with increasing physical activity in this participant group and achieving long term behavior change, reducing patient symptoms as well as reducing the cost to the National Health Service (NHS).

## 1 Introduction

Diabetes affects 4.7 million people in the UK (1) and costs the NHS approximately £10 billion per year (2). Diabetes is recognized as a global epidemic (3) and is increasing year on year (4).

Increases in exercise and physical activity are commonly used in people with type 2 diabetes as an initial method for those newly diagnosed (5), in addition to maintenance and control of symptoms (3),and also have notable health benefits for people with type 1 diabetes (6). Yet despite this intervention being cost effective and potentially easy to implement, there is a lack of consensus in appropriate prescription of physical activity or exercise for patients with diabetes, and research on exercise interventions lacks consistency in terms of exercise modality and duration (3, 7). While patients may be encouraged to be more active by their healthcare provider, detailed and condition-specific advice to support this is limited (8) which may lead to ineffective prescription of exercise and reduce the likelihood of the patient making long-term lifestyle changes. In addition to having a limited impact on the progression of the condition, this also implies an associated cost in terms of medication and health care for the long-term management of the patient with diabetes, as well as potentially limiting their quality of life (3).

The aims of this research are to evaluate the goals and outcomes for providing exercise interventions for people with diabetes, and to establish which type of intervention(s) is considered most beneficial.

## 2 Methods

### 2.1 Rationale

A Delphi study is a method for attempting to obtain consensus between experts and stakeholders using a series of ‘rounds’ of questions. Delphi studies gain information from a wide range of stakeholders in an iterative and anonymous way (9).

People with diabetes may be provided with physical activity recommendations from pre-diagnosis through to managing advanced symptoms such as peripheral neuropathy, by healthcare professionals as well as fitness professionals, so a Delphi study to attempt to establish consensus within this range of professions is appropriate to gather opinion from a diverse group.

### 2.2 Study design and development

Informed consent was obtained prior to participation, and ethical approval was granted by Anglia Ruskin University research ethics panel. A three-round approach was adopted as this is considered optimal to generate consensus (10). The surveys were delivered online (www.onlinesurveys.ac.uk) with links sent to participants via email, and data was gathered between April and September 2021.

This study has been reported considering the methodological considerations presented in the CREDES checklist (11) which is included in the **Supplementary Material**.

### 2.3 Qualitative round

A classical approach was used for the first round of the Delphi study. As previously noted, research on exercise interventions in people with diabetes lacks clear consensus, so using a literature review to establish the initial topics would be likely to miss key factors from the participants’ professional experience. To ensure that this was fully represented, free-text responses were gathered on the aims of intervention and type of exercise that would be most appropriate (12). In addition, participants were asked for any general comments. These responses were then collated following an inductive thematic analysis (13) to identify key themes from the panel to present for consensus.

All suggestions were retained to ensure a breadth of response and to reduce bias due to over representation of any specific group. Specific themes for future rounds were kept in the participants own words where possible to prevent ambiguity or confusion, and to help with readability for the participants of these questions in future rounds.

### 2.4 Quantitative rounds

Each survey was pilot tested using both healthcare and fitness professionals to improve the structure of the survey and to ensure that the statements were understandable by the target audience. As a result of this pilot testing, a ‘Don’t know’ option was added to each item to account for various levels of experience and technical knowledge that may exist between different professions. This also prevented any attrition due to the participants perceived lack of knowledge or due to more dominant individuals (14). During each survey, participants were encouraged to add comments if they wanted to revise the subsequent questions in later rounds to avoid any ambiguity.

The second round presented the themes established in the first round with a five-item Likert scale to rate the perceived importance of each aspect. Consensus was defined at 70% of all participants stating ‘Strongly Agree’ or ‘Agree’, which is consistent with other Delphi studies (15).

Any items that had not reached consensus following the second round were presented to the participants again in round three, alongside a summary of how previous voting had rated each aspect to allow each participant to consider the overall view before providing a new rating (16). Previous voting was presented in simple percentage values so that participants did not need any specific knowledge to interpret any measures of central tendency or variance, making this accessible and easy to understand (17). If consensus for an item was already achieved in round two, then this item was removed from rescoring. Participants were blinded to each other’s responses other than in this collective summary to reduce any bias (15).

Participants were given six weeks to complete each round, and regular reminders were sent via email to encourage ongoing participation. The estimated time to complete each round was 15 minutes for round one, and 10 minutes for rounds two and three. There were no incentives provided for participation. All participants that took part in the original round were asked to complete subsequent rounds to attempt to gain a complete reflection from the original panel and to avoid invalid consensus due to drop out or attrition (18).

### 2.5 Expert panel

Participants were included if they were UK-based healthcare or exercise professionals that considered themselves experienced in prescribing exercise interventions for people with diabetes. Participants were purposively sampled to ensure a range of different experience on the panel, and included physiotherapists, podiatrists, yoga teachers, GP referral exercise instructors, endocrinology consultants and diabetes specialist nurses to gain a wide representation across a number of specialist areas (16). Recruitment of panelists was done via social media, personal contacts and using snowball sampling to increase the diversity of the included sample population (19).

No limitations were placed on what level of qualification or years of experience would denote an ‘expert’ to ensure that anyone with any relevant contribution could participate (20). The choice to include both healthcare and fitness professionals was made to ensure that intervention advice given early in diagnosis (for example via GPs or practice nurses) was included rather than just considering those patients that are advised via physiotherapy or referral interventions (21). This breadth of participant roles ensures advice at each stage of diagnosis and treatment is represented in the panel where possible. Participants were limited to UK-practicing professionals to limit any impact of discrepancy in healthcare that may exist in other countries, and to ensure consistency of responses.

It has been suggested that a sample size of 12 is valid to ensure consensus within a Delphi study, with larger sample sizes leading to diminishing returns (22). Attrition within Delphi studies has been found to be variable with as low as 45% response rates recorded in some studies (23). This level of attrition was also expected to be impacted by the COVID pandemic. An initial sample size of 45 participants was selected for use in this study, to allow for a comparable sample to previous Delphi studies, whilst anticipating the possibility of 50% attrition in later rounds (22). The responses of both healthcare and fitness professionals were monitored to ensure that the participation of each group remained consistent, and to reduce attrition bias if those with minority opinions withdrew from the study (23).

## 3 Results

### 3.1 Participant demographics

Of the 45 participants recruited, thirty provided demographic information. The majority of participants in the study were female (77%). In addition, most of the participants that offered demographic information were educated to degree standard or above (67% of fitness professionals, 81% of healthcare professionals). In each round there was a consistent split between healthcare and fitness professionals, which suggests that the results are appropriately representative of each sector.

### 3.2 Attrition

Of the 45 participants that contributed to the initial round, 26 (58%) agreed to participate in further rounds. Participants did not always provide a reason for drop out, but the ongoing COVID situation in the UK during the time that the surveys were open accounted for some attrition due to redeployment of healthcare and fitness professionals resulting from lockdown and the vaccine rollout. Of these 26 participants, 21 participated in round 2 (47%) and 19 in round 3 (42%).

### 3.3 Survey development

An inductive thematic analysis of the free text responses resulted in 25 statements relating to the goals and aims of exercise interventions, 11 statements relating to provision (for example statements on cost or accompanying services) and 18 statements on the exercise modality. These statements can be found in **Table 1**. These statements were all presented in the surveys in alphabetical order to avoid any leading questions or biased responses based on the order in which this was viewed by participants. In addition, healthcare professionals are less motivated to respond to surveys where clinical items dominated (23), so using alphabetical order ensured that these were potentially spread throughout the presented options. Participants could respond with ‘don’t know’ in each case if they were unsure or the concept was outside their scope of practice or experience.

**Table 1:** Statements and percentage agreement in each round.

	Round 2 (% agree or strongly agree)	Round 3 (% agree or strongly agree)
Aims of intervention		
Achieve or maintain a healthy weight	95.24	
Break down barriers to exercise participation	95.00	
Improve balance	78.95	
Improve circulation	90.00	
Improve glycemic control	95.00	
Improve muscle tone	90.00	
Improve neuropathy	47.06	64.71
Improve sleep patterns	78.95	
Increase bone health	84.21	
Increase claudication distance tolerance	93.33	
Increase confidence	95.00	
Increase education or awareness	90.00	
Increase physical activity	94.74	
Increase psychological wellbeing	94.74	
Achieve long term behavior change	95.00	
Maintain CV health	95.00	
Maintain joint ROM	80.95	
Manage insulin resistance	85.00	
Meet recommended guidelines	88.24	
Reach their patient centered goals	78.95	
Prevent “hypos”	47.37	47.06
Gain/maintain social support	85.00	
Reduce falls	84.21	
Reduce fear of exercising	88.89	
Reduce secondary conditions	84.21	
Provision		
Are accessible to those with visual impairment or speakers of other languages	95.00	
Are open ended (without a specific end date)	76.47	
Are supervised activities	47.37	36.84
Have options for those completely new to exercise	95.00	
Include individual tailoring	90.48	
Only last for 30 mins or less	20.00	5.88
Only last for 30 mins or more	20.00	17.65
Provide advice on foot care	75.00	
Provide diet advice	85.00	
Provide free gym and/or exercise activity	75.00	
Provide information about medication and how the interaction may affect this	89.47	
Modality		
Activities of daily living	95.00	
Aerobic exercise	85.00	
A mix of low and high intensity exercise	65.00	84.21
Breathing exercises	60.00	44.44
Brisk walking	80.95	
Chair based exercise	70.00	
Cold showers	7.69	12.50
Contact sports	29.41	5.88
Core strength	89.47	
Cycling	57.89	63.16
High impact activity	40.00	33.33
High intensity exercise (e.g. sprinting or maximal weight lifting)	30.00	42.11
Meditative walking	57.89	63.16
Resistance training	77.78	
Strength and balance (falls prevention) exercise	90.00	
Swimming	73.68	
Weightbearing exercise	70.00	
Yoga or Pilates	80.00	

### 3.4 Round 2 results

In round 2, the majority of items (89%, n=16) relating to goals and aims reached the 70% consensus limit. No items were removed, revised or added as a result of the participants’ comments. The two items that failed to reach the 70% consensus level were the aim of the intervention to improve neuropathy and to prevent “hypos” (hypoglycemia or low blood sugar) both achieving 47% consensus.

Within the section on provision, consensus was not achieved when considering whether activities should be supervised (47% agree or strongly agree) and when considering duration. Within the qualitative round, there was a difference of opinion on duration with some participants stating that only less than 30 minutes duration was appropriate, while others stated it had to be over 30 minutes to be effective, so both options were presented in round 2 to attempt to achieve consensus. In each case, most participants stated ‘Neither agree nor disagree’.

When considering exercise modality, the majority of participants agreed that both aerobic (85%) and resistance exercise (78%) were important activities for a person with diabetes. In addition, some specific exercise modalities such as swimming, yoga and Pilates, brisk walking, core strength and strength and balance (falls prevention) exercises also reached consensus from the panel. Eight other modalities failed to reach the prescribed consensus level and were therefore presented to the panel again in round 3.

### 3.5 Round 3 results

One item, to include a mix of high and low impact activity, reached consensus following round 3. The remaining items were discarded at this point due to not reaching consensus.

**Figure 1:**
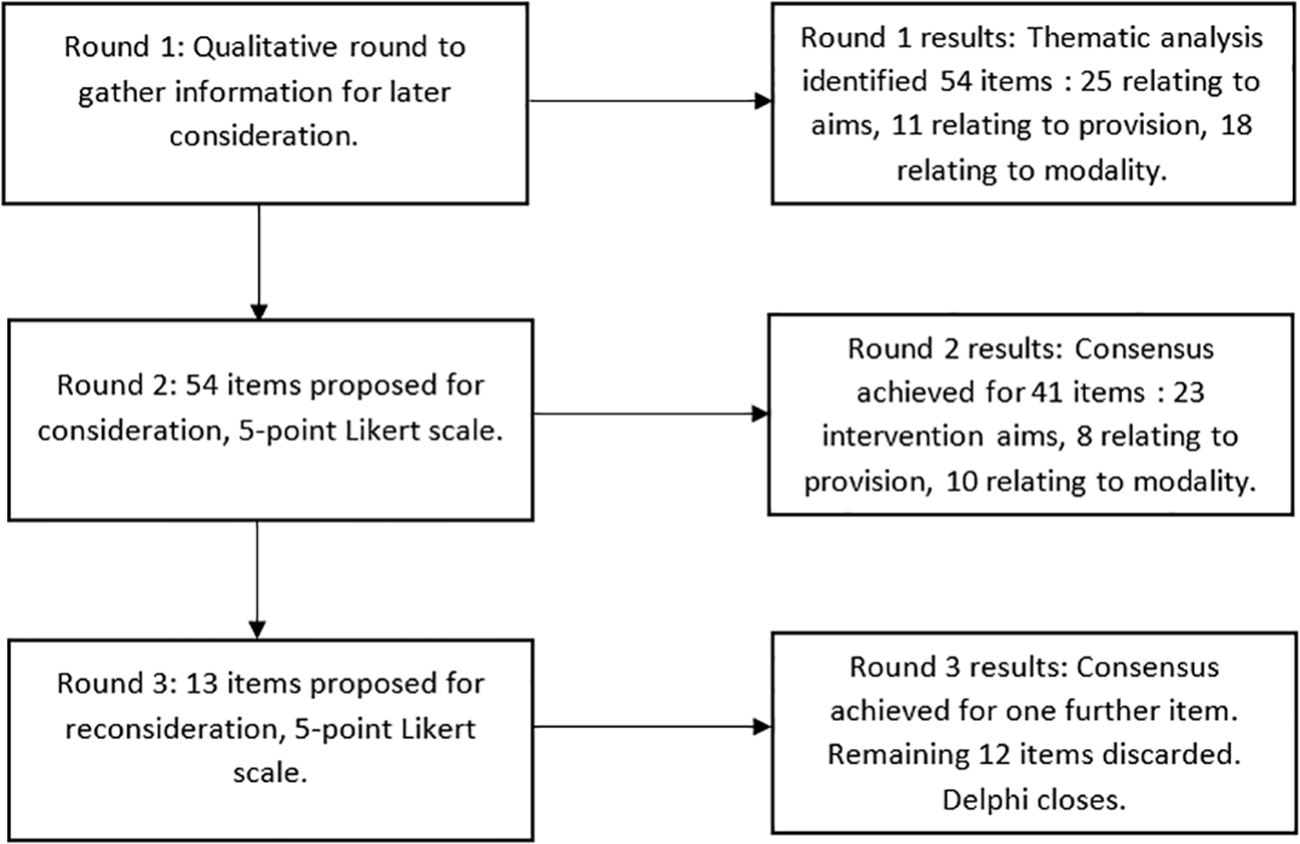
Flowchart illustrating the Delphi process.

The progression through the different rounds and consensus reached is presented in **Figure 1**.

The statements and responses from each round are presented in **Table 1**.

## 4 Discussion

The aim of this study was to evaluate the level of consensus in exercise prescription for people with diabetes. Previous evidence suggested that people with diabetes may struggle to get reliable recommendations from their healthcare professionals (24). In this study, we gathered data about the exercise and physical activity prescription within healthcare and fitness settings in the UK to show specific similarities or differences in practice. Although most of the items achieved consensus, these are often contradictory and include many different modalities. This supports the aims of the intervention as being patient-led and tailored, as different exercise modalities may be appropriate at different stages of the diagnosis.

Despite finding a lack of consistent recommendation between health and fitness professionals in providing advice and exercise prescription, the use of physical activity to manage diabetes symptoms and improve the overall health of people with diabetes is recommended as part of a multifactorial approach to treating the condition (25). In particular the American College of Sports Medicine have recently adapted their recommendations for people with diabetes to specifically include physical activity, focusing on reducing sedentary time and moving more rather than advocating participation in planned, structured exercise (26). This is especially important in managing diabetes due to a high level of inactivity (27) with this patient population being disproportionately sedentary (28). While healthcare professionals may be familiar with providing general diet and medication advice, they may lack confidence when promoting physical activity and exercise (29).

Of the original 25 items presented in round 2 to establish the aims of the interventions, the majority reached consensus. Where consensus was not achieved this may be a lack of shared knowledge and understanding within the participants, as preventing hypoglycemia may not be a concern for those patients prescribed metformin, for example, and it may not be possible to ‘improve neuropathy’ rather than alleviate the symptoms (30).

When considering aspects of provision that could be useful, consensus was achieved on most options that related to increasing accessibility, such as reduced cost and options for those new to exercise. The lack of consensus on duration of activity, with no specific time for activity being preferred more than others may relate to the aim for a ‘person centered’ approach, with some durations being more appropriate for those new to exercise or older, and others being more applicable to other demographics (31). The need for supervised activity did not reach consensus, which may relate to the prescription of general physical activity rather than exercise interventions. However, a recent study has suggested that the presence of an instructor caused increased adherence and long-term behavior change in overweight adults (32) so this may be an area requiring further consideration.

When selecting an expert panel for the Delphi study, any professional with experience of prescribing exercise or physical activity were included, as this would represent a range of ability and knowledge that could contribute to our attempts to reach consensus. However, professionals within this group may see people with diabetes at different stages of their condition. As mentioned previously, nurses are likely to advise patients with diabetes from diagnosis onwards, whereas physiotherapists may not become involved until patients are referred with comorbidities (21). When considering interventions, cost also becomes a factor with healthcare professionals providing their services for free via the NHS, whereas a personal trainer is associated with an additional cost that may not be accessible. Similarly, there is an additional cost associated with yoga teachers and gym instructors represented on the panel, which may influence their recommendations by needing to provide ‘value for money’ with specific exercise prescription. This difference of opinion between professionals that a person with diabetes may approach for advice is inevitably confusing and frustrating (24) and may discourage patients from becoming more active.

The lack of identification of a small group of preferred exercise modality found in this study is supported by the aims of the interventions provided by the participants, with some of the additional free-text comments suggesting that the type of exercise intervention is less important than creating an overall long-term behavior change and ongoing increased activity. While some participants were very specific in their recommendations and content, this was not the case for the whole cohort. One of the key ideas mentioned in the first round was the idea of tailored, patient specific interventions which would override the need for a “one-size-fits-all” diabetes intervention. Diabetes affects a wide range of ages, and this can also affect the appropriateness of prescribed interventions. In addition, comorbidities and complications as a result of having diabetes can also restrict the interventions that could be considered appropriate. Although advocating long term behavior change as an important factor, it is not clear how this will be delivered in practice, with available schemes such as exercise referral lacking clear focus on behavior change (33), and practitioners lacking both leadership and self-awareness to deliver interventions supporting behavior change (34). While compliance with recommended levels of physical activity is a factor in managing long term change (35) ensuring this is achieved in practice is problematic. In particular, structured provision only provides marginal benefit over other interventions (such as providing basic advice) (36) supporting the overall emphasis on unstructured physical activity via activities of daily living found within this Delphi study. This is also supported by recent advice from the ACSM advocating reducing sedentary behavior to manage symptoms and slow progression of diabetes (26).

### 4.1 Strengths and limitations

A key strength of this research is the breadth of participant experience within the expert panel, to ensure a diverse range of opinion was gathered to attempt to achieve consensus. Despite attrition, an appropriately sized panel was retained for all three rounds.

Due to recruitment being via social media and snowballing, there may be some sampling bias due to the participants already having an interest in non-medical interventions to treat people with diabetes. In addition, due to the constructivist nature of Delphi studies (11) and the lack of consistency and consensus within existing literature it is possible that this study contains information that is not present in existing literature, or that existing literature presents features not considered in the results of this study.

### 4.2 Clinical implications

The lack of consistent preference for exercise aim and modality found in this study suggests that exercise prescription may be inconsistent, and the lack of clear recommendations may discourage patients to become more active. Lack of knowledge or experience in exercise prescription can be overcome by providing general physical activity advice, and signposting to relevant resources such as the ACSM statement (26) and Diabetes UK resources (37). In this way, clear, unambiguous guidelines from both fitness and health professionals can encourage people with diabetes to make long term changes to their health behavior, managing their condition and reducing cost to the National Health Service.

## 5 Conclusion

While the benefits of increased physical activity within this population group are widely advocated, the current advice provided may be confusing and contradictory. The large amount of consensus on different intervention methods achieved in this study supports this, and more research is required on the consistent and specific promotion of physical activity advice from healthcare and fitness professionals and how to achieve long term behavior change in this participant group.

## Data availability statement

The raw data supporting the conclusions of this article will be made available by the authors, without undue reservation.

## Ethics statement

The studies involving humans were approved by School of Psychology and Sport Sciences ethics committee at Anglia Ruskin University. The studies were conducted in accordance with the local legislation and institutional requirements. The participants provided their written informed consent to participate in this study.

## Author contributions

CS: Conceptualization, Formal analysis, Investigation, Methodology, Validation, Visualization, Writing – original draft. FC: Writing – review & editing. MAT: Writing – review & editing. AM: Conceptualization, Methodology, Supervision, Visualization, Writing – review & editing.
